# Transcatheter arterial microembolization for refractory chronic pain associated with familial gouty tophi: a case report with 2-year follow-up

**DOI:** 10.1186/s42155-026-00701-y

**Published:** 2026-05-21

**Authors:** Eiji Sugihara, Motoki Nakai, Yuki Takara, Toru Saguchi, Taro Tanaka, Kazuhiro Saito, Kenji Endo

**Affiliations:** 1 Department of Radiology, Tokyo Medical University, 6-7-1 Nishishinjuku, Shinjuku-Ku, Tokyo, 160-0023 Japan; 2 Department of Orthopaedic Surgery, Tokyo Medical University, Tokyo, 160-0023 Japan

**Keywords:** Transcatheter arterial microembolization, Gouty tophi, Imipenem/cilastatin, Chronic pain, Musculoskeletal IR

To the Editor,

Chronic pain associated with gouty tophi can be refractory to standard pharmacotherapy [1] and significantly impair quality of life. Recently, abnormal neovascularization associated with chronic inflammation has gained attention as a potential pain generator. We report a case of refractory familial gouty tophi with a 40-year history, successfully treated with transcatheter arterial microembolization (TAME), with a focus on quantitative clinical outcomes over a 2-year follow-up.

A 60-year-old male presented with a 40-year history of gout and monthly flare-ups in both halluces. His family history was notable for hyperuricemia in his father and all six siblings. Despite intensive medical therapy, including colchicine (1 mg/day) and febuxostat (20 mg/day), he experienced severe pain during attacks, with a Numerical Rating Scale (NRS) score of 9/10. Contrast-enhanced MRI (Fig. 1) and angiography (Fig. 2a and Fig. 3a) revealed hypervascularity (vascular blush) corresponding to the tophaceous lesions at the right first interphalangeal (IP) and left first metatarsophalangeal (MTP) joints.

**Fig. 1:**
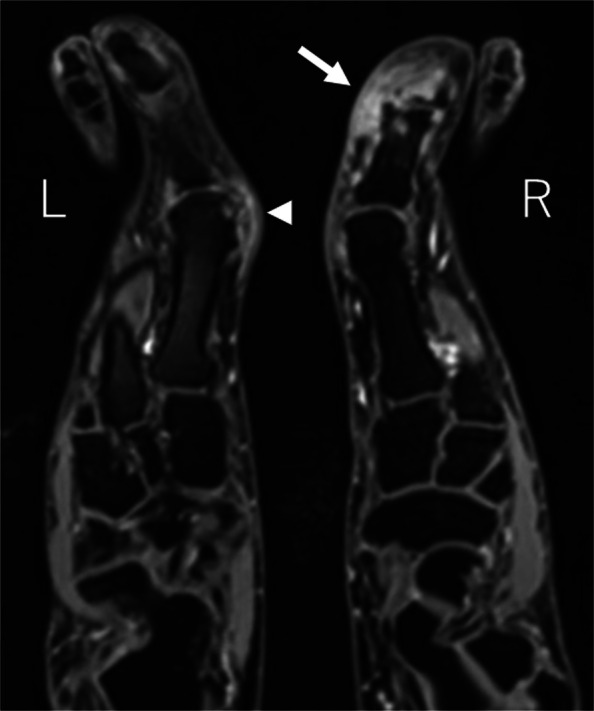
Contrast-enhanced MRI showing hypervascular tophi at the right first IP joint (arrow) and left first MTP joint (arrowhead)

**Fig. 2:**
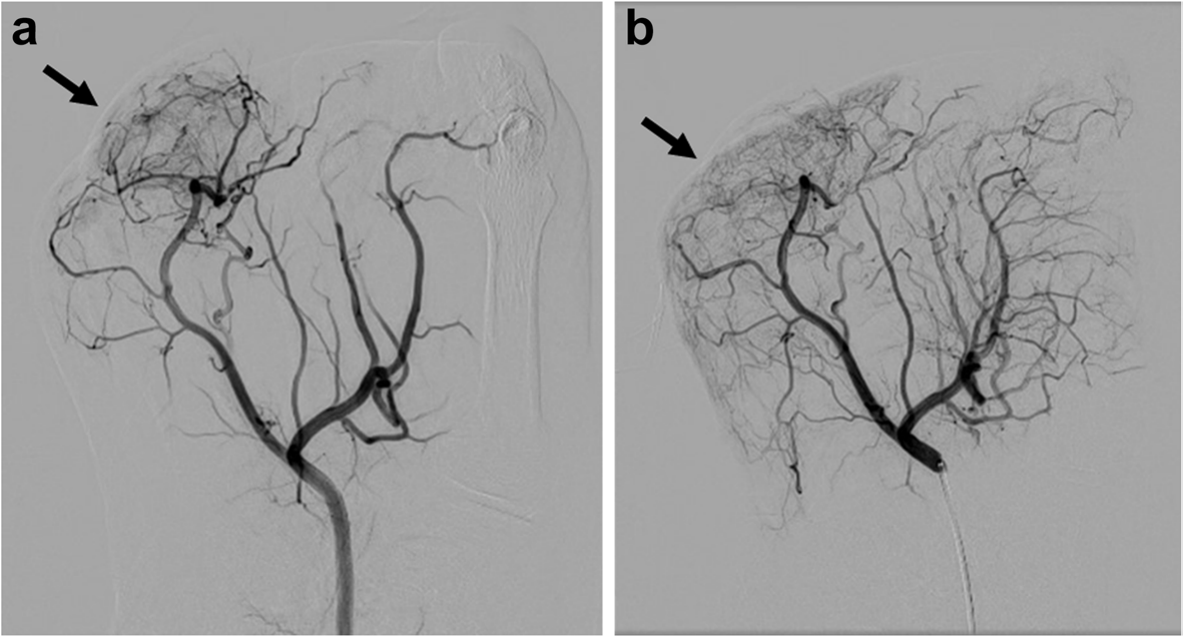
Right dorsalis pedis angiography: **a** vascular blush at the right first IP joint; **b** disappearance after 2.5 mL IPM/CS injection

**Fig. 3:**
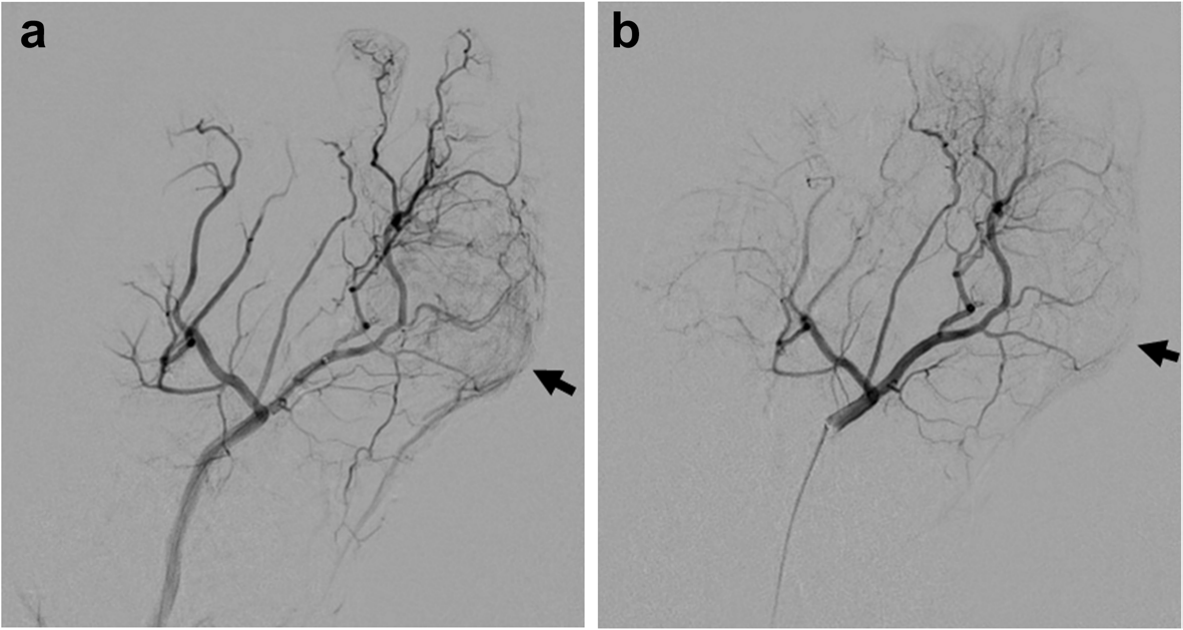
Left dorsalis pedis angiography: **a** vascular blush at the left first MTP joint; **b** disappearance after 2.5 mL IPM/CS injection

TAME was performed under local anesthesia alone, without sedation. A 2.6-Fr microcatheter (Tokai Medical Products, Aichi, Japan) was used to deliver imipenem/cilastatin (IPM/CS) microparticles (0.5 g suspended in 10 mL nonionic contrast medium, Iomeron 300; Bracco, Milan, Italy) into the dorsal metatarsal arteries (Fig. 2b and Fig. 3b). For the right and left procedures, the dose area product (DAP) was 8.76 and 14.33 Gy·cm^2^, the total air kerma was 50.8 and 90.2 mGy, and the fluoroscopy time was 20.4 and 20.6 min, respectively.

Following treatment, the patient showed marked clinical improvement. The frequency of flare-ups decreased from 1–2 episodes per month at baseline to approximately one episode every 6 months during the 2-year follow-up. Inter-attack pain remained minimal, with an NRS score of 1/10. Notably, the daily colchicine dose was reduced from 1 mg/day to 0.5 mg/day, while the febuxostat dose (20 mg/day) remained unchanged. Serum uric acid levels remained stable throughout the follow-up (baseline: 6.8 mg/dL; 1 year: 7.2 mg/dL; 2 years: 6.8 mg/dL), suggesting that the clinical improvement was not attributable to altered systemic urate metabolism.

The clinical efficacy of TAME for refractory musculoskeletal pain has been increasingly reported [2–5]. In this case, the marked clinical response suggests that disruption of the pathological neurovascular unit within the tophi may have contributed to pain relief. By eliminating fragile abnormal vessels, TAME likely reduces nerve stimulation and the leakage of pain-inducing cytokines [6, 7]. In this case, the significant reduction in pain and medication requirements despite stable uric acid levels supports the hypothesis that TAME directly targeted the local pain-generating mechanism rather than systemic metabolic factors. The temporary nature of IPM/CS may also contribute to a favorable safety profile, particularly in distal extremities [8].

While limited to a single case, these findings suggest that TAME may represent a safe and potentially effective minimally invasive option for patients with refractory gouty pain. Further studies are warranted to validate these observations.

## Data Availability

The data supporting the findings of this case report are included within the article.
